# Gastric Cancer With Multiple Bone Metastases: An Uncommon Primary Presentation

**DOI:** 10.7759/cureus.29467

**Published:** 2022-09-22

**Authors:** João Barbosa-Martins, Salomé Marques, Olinda Miranda, Bárbara Lima, Jorge Cotter

**Affiliations:** 1 Medical Oncology Department, Hospital da Senhora da Oliveira, Guimarães, PRT; 2 Internal Medicine Department, Hospital da Senhora da Oliveira, Guimarães, PRT

**Keywords:** thrombocytopenia, bone marrow infiltration, signet ring cell, bone metastasis, gastric cancer

## Abstract

Gastric cancer (GC) is a worldwide health condition of major concern, with gastric carcinoma with signet ring cell features being increasingly reported. A 61-year-old woman was admitted to the Emergency department with back pain, gastrointestinal complaints, and weight loss. A lumbar and hip computed tomography (CT) was performed and revealed multiple suspicious secondary bone lesions. Laboratory test results reported anemia, thrombocytopenia, and elevated alkaline phosphatase. On thoracic-abdominal-pelvic CT, multiple bone lesions suggestive of metastases were visible on the vertebral spine, ribs, pelvic bones, and proximal femurs, but no identifiable primary or visceral lesions were described. Upper endoscopy identified a gastric adenocarcinoma, and both gastric and bone lesions, especially bone lesions, contained a relevant amount of signet ring cells. The patient was referred to the Medical Oncology department, however, her condition evolved unfavorably. GC with restricted bone metastasis is rare at presentation and has a poor prognosis. Despite its infrequency, clinicians should consider GC involvement when evaluating secondary suspicious bone lesions.

## Introduction

Worldwide, gastric cancer (GC) is a leading cause of cancer-related death, with a median survival time of less than 12 months in advanced stages [1]. Its incidence has been decreasing, however, Portugal still has a high rate of GC [2]. Despite the global decrease in GC incidence, newly diagnosed cases of gastric carcinomas with signet ring cell features have been increasing [3]. Signet ring cells are thought to be an intermediate stage between squamous and adenocarcinoma cells or perhaps a glandular or mucin-secreting component of a squamous cell carcinoma [3]. Like in all cancers, it is crucial to assess the presence or absence of metastases in order to offer the best treatment available [4]. GC metastases are more frequently found in lymph nodes, peritoneum, or liver, while bone metastases are uncommon at initial presentation [5,6]. Here, we present an uncommon case of GC with disseminated bone and bone marrow infiltration, without any other metastatic sites.

## Case presentation

**Figure 1:**
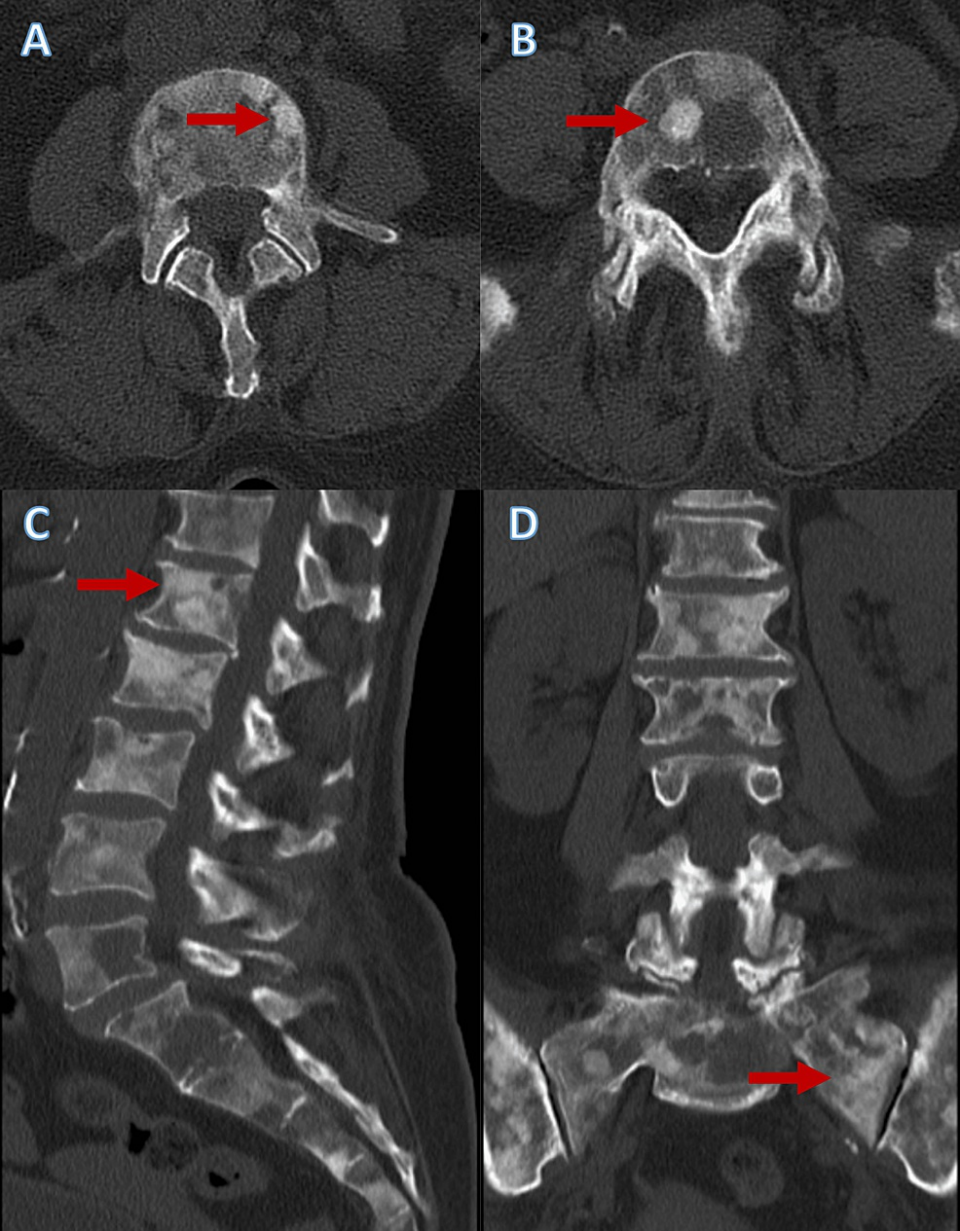
Suspicious vertebral bone lesions Lumbar computed tomography revealed diffuse heterogenety of the lumbar vertebrae and sacrum bone marrow (A, B, C, and D), secondary to malignant infiltration (examples highlighted by the red arrows)

**Figure 2:**
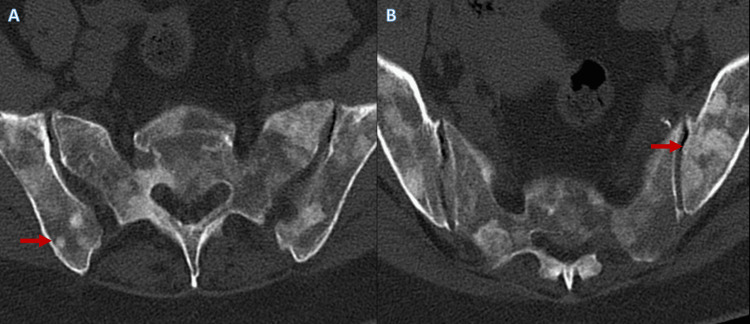
Suspicious hip bone lesions Computed tomography of the hip revealed multiple bilateral bone lesions (A and B), secondary to malignant infiltration (examples highlighted by the red arrows)

A 61-year-old woman presented to the Emergency department with a three-week history of lumbar pain. The pain was moderate-to-intense, continuous, with mechanical characteristics, and radiated to the pelvis and right shoulder. She also reported having scarce episodic melaenas, anorexia and weight loss in the previous month, and long-term dyspepsia. She had a history of hypertension and a positive cervical test for human papillomavirus but no cytological alterations. At physical examination, she presented with no fever, blood pressure of 144/80 mmHg, heart rate of 100 beats/minute, and oxygen saturation of 100% on room air. She had no palpable lymph nodes and no alterations to pulmonary or cardiac auscultations. The abdomen was painless, and she had no neurological deficits. She had a spine and hip X-ray, which revealed suspicious bone lesions. Then, a hip and lumbar computed tomography (CT) was performed, which showed diffuse heterogeneity of the vertebral bone marrow suggestive of secondary infiltration (Figure 1) and multiple hip bone lesions (Figure 2).

**Figure 3:**
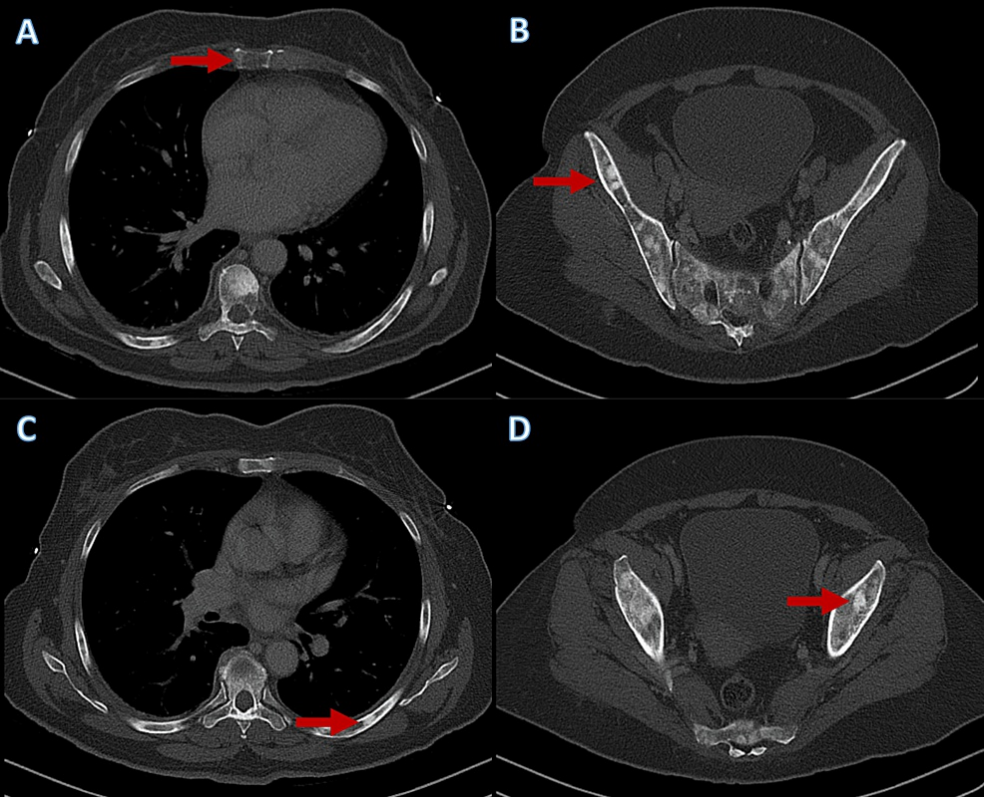
Multiple skeletal metastases Chest, abdominal, and pelvic computed tomography revealed multiple blastic and lytic lesions at the sternum (A), hip (B), ribs (C), and proximal femurs (D), compatible with diffuse bone metastases (examples highlighted by the red arrows)

The laboratory test results revealed the presence of normocytic normochromic anemia with a hemoglobin level of 7.0 g/dL (reference range within our center: 12.0-16.0 g/dL); leukocyte count of 7.1x10^3^/mL (4.8-10.8x10^3^/mL); lymphocyte count of 1.6x10^3^/mL (1.0-4.8x10^3^/mL); and thrombocytopenia, with a platelet count of 98x10^3^/mL (150-350x10^3^/mL). She presented normal iron levels and normal total iron binding capacity, high ferritin of 1074.5 ng/mL (10-291 ng/mL), and the peripheral blood film revealed erythrocyte and platelet anisocytosis. She presented no folic acid or cobalamin deficiency, no hypercalcemia, and had normal prothrombin and activated partial thromboplastin times. She had slightly elevated liver enzymes, with aspartate aminotransferase (AST) being 69 IU/L and alanine aminotransferase (ALT) being 81 IU/L (15-37 and 30-65 IU/L, respectively) and also elevated C-reactive protein (CRP) of 37.3 mg/L (<3.0 mg/L). The alkaline phosphatase (ALP) and lactic acid dehydrogenase (LDH) levels were elevated with values of 2231 IU/L (46-116 IU/L) and 372 IU/L (120-246 IU/L), respectively, and the carcinoembryonic antigen (CEA) levels were also high with values of 803 ng/mL. A chest, abdominal, and pelvic CT with intravenous contrast media was performed, which revealed multiple lytic and blastic lesions in the vertebral column, ribs, hip, and bilateral proximal femurs, suggestive of diffuse bone metastases (Figure 3).

**Figure 4:**
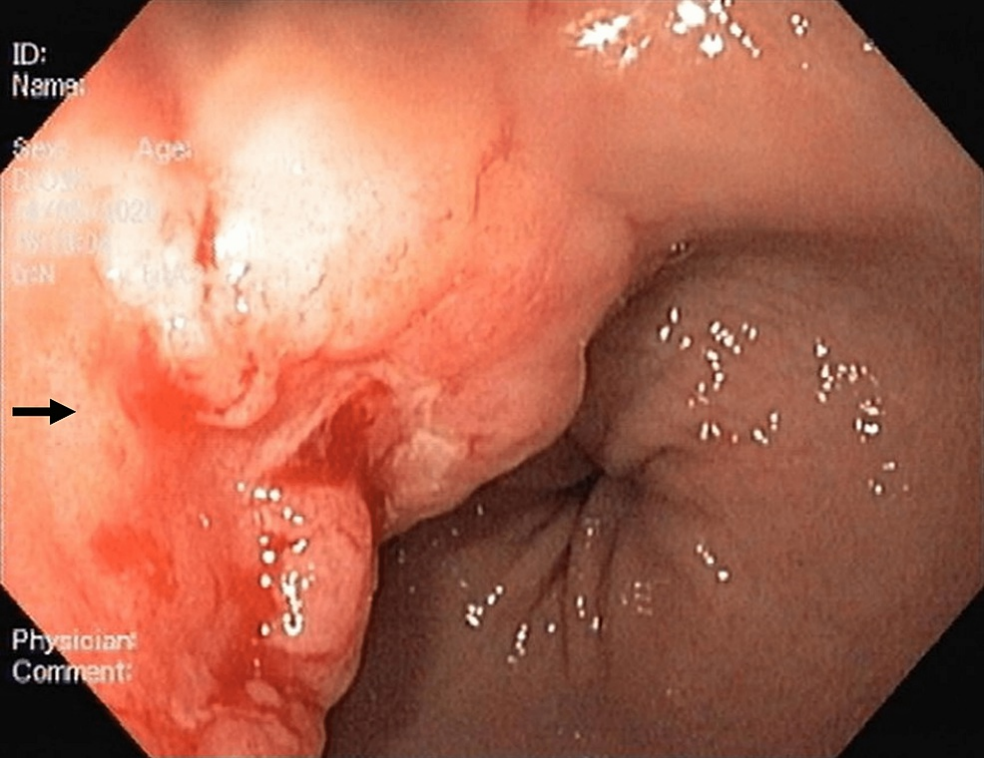
Suspicious gastric lesion Upper endoscopy showed a neoplasia of about 40 mm with ulceration areas, located at the anterior wall and proximal antrum lesser curvature (black arrow)

No secondary lymph nodes or other suspicious lesions were reported in any other organ or system. The patient was admitted for further analysis, received analgesia and supportive treatment, and was placed in a Jewett brace for pain control. The etiological investigation revealed no monoclonal peaks at serum protein electrophoresis and the peripheral blood immunophenotyping did not reveal any signs of malignancy. Breast mammography and thyroid echography were unremarkable. The upper endoscopy revealed a 40 mm ulcerated neoplasia on the anterior gastric wall and proximal antrum lesser curvature (Figure 4).

**Figure 5:**
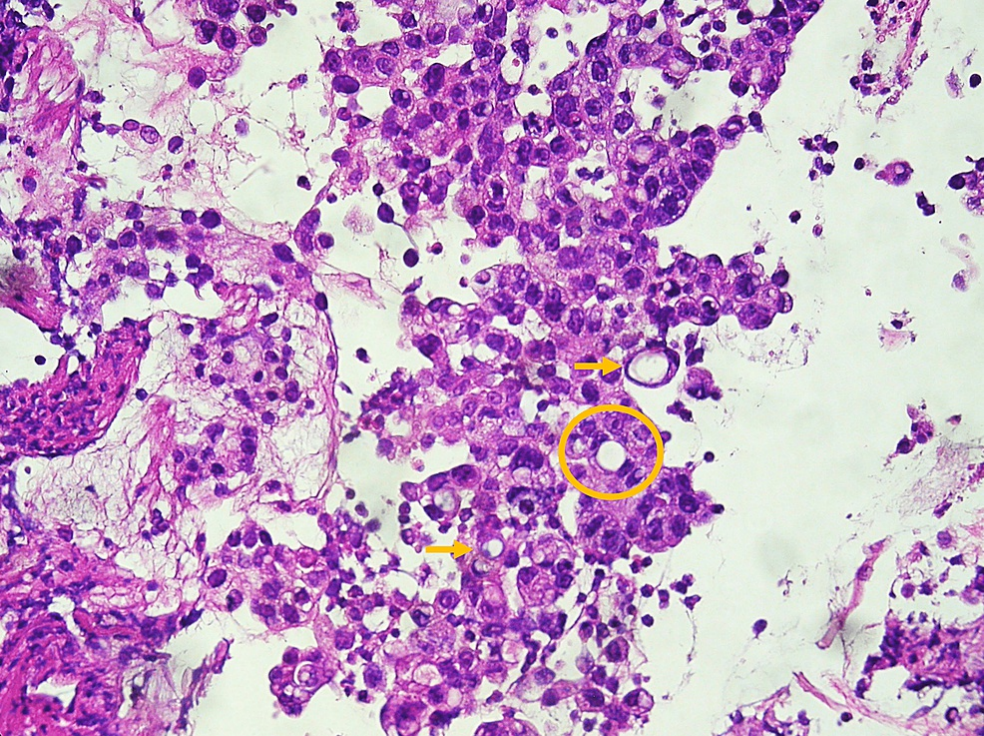
Gastric lesion biopsy Mixed adenocarcinoma with areas of tubular differentiation (example in the yellow circle) and areas of poorly cohesive cells, including signet ring cells (examples highlighted by yellow arrows) Hematoxylin-eosin staining, 40x magnification

**Figure 6:**
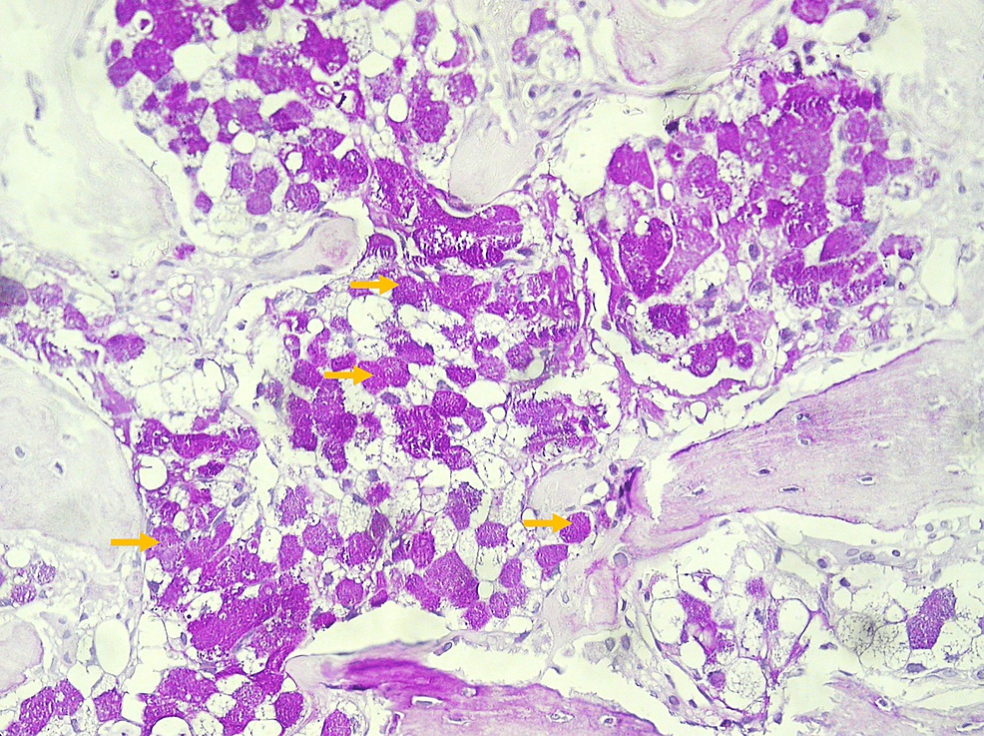
Bone biopsy of an iliac lesion Bone trabecular tissue involved by a metastatic lesion, with characteristics of adenocarcinoma with signet ring cells (examples highlighted by yellow arrows) Periodic Acid-Schiff (PAS) staining, 40x magnification

An HER-2 negative adenocarcinoma with areas of tubular pattern and signet ring cells was confirmed on gastric biopsy (Figure 5). A bone biopsy of an iliac lesion revealed a secondary metastatic lesion with features of adenocarcinoma with signet ring cells (Figure 6).

She was examined by the Medical Oncology department and proposed for a multidisciplinary consultation. During her admission to the hospital, the pain and ongoing cytopenia became challenging to manage and her condition deteriorated, resulting in a fatal outcome on the 60^th^ day of admission.

## Discussion

Notwithstanding the fact that GC incidence and mortality have been decreasing since the early 20^th^ century, it remains an important worldwide cancer burden [1,7]. In symptomatic patients, the clinical presentation may include weight loss, vomiting, dyspepsia, dysphagia, early satiety, and/or iron deficiency anemia [4]. In the present case, the diagnosis was reached by assessing the etiology of the patient’s back pain. GC metastases are typically found in the liver, peritoneum, and lymph nodules and less frequently in the ovaries, lungs, central nervous system, spleen, adrenal glands, and bone [5,6]. GC less frequently invades the bone (0.9-13.4%) [8] or the bone marrow (0.024%) [7], and this state is usually described as the disease progresses and less as the primary presentation [9,10]. Moreover, in one of the largest multicentric works in which GC bone metastasis onset was detailed, only 28% presented these lesions at the time of diagnosis [10]. The incidence of GC bone metastases may vary with the diagnostic method, to the point that some studies suggest it may be underestimated [11]. In fact, screening of bone lesions may be recommended in other neoplasias but not routinely in GC [8,11]. Its presentation may be synchronous or metachronous, and the lesion type may be osteolytic, osteoblastic, or mixed [8]. Typically, when the bone is involved, the most reported sites are the spine, pelvic bones, extremities, ribs, sternum, scapula, and skull [8]. Anatomical features might explain how GC metastasizes to the bone, as a portion of the bloodstream from proximal stomach regions may drain to the Batson venous plexus, which ends in penetrating the spine via the azygous and hemiazygos veins [12]. Bone involvement is more probable in superficially depressed, ulcerated, large, or corpus carcinomas and is more often associated with the presence of lymph node metastasis and elevated CEA levels [13]. The most likely etiologies may be poorly differentiated signet ring cells and high-grade and mucinous carcinomas [8,13-15]. Although infrequent, this pattern of GC metastasization might be observed in younger patients and is associated with a worse prognosis [8]. The case we present was a patient with a primary gastric mixed adenocarcinoma with tubular areas and poorly cohesive cells, including signet ring cells, which presented uncommon mixed and synchronous exuberant bone metastases, of the signet ring cell component, in several skeletal segments. She had high CEA levels but no other structures affected at the time of diagnosis. In fact, metastases confined to bone are uncommon in GC, as demonstrated by Wen et al., who reviewed 66 GC patients with bone metastases, and only 10 had no concomitant extraosseous involvement [11]. Different from our case, GC bone metastasis may also manifest as spinal compression, pathologic fractures, and hypercalcemia [10,11].

Bone marrow involvement, although rare, is more found in GC among solid tumors, and not only has it a poorer prognosis but is also more frequent in patients in their fifth decade of life, with gastric carcinomas with poorly cohesive cells and/or signet ring cells and with extensive bone metastasis [5,9,16-19]. The most typical hematologic abnormality observed in these patients is thrombocytopenia [5]. Most of these features were also present in our patient, however, differently from our case, bone marrow metastasis may also present as intravascular disseminated coagulation, microangiopathic hemolytic anemia, thrombosis, and leukoerythroblastic reaction [19].

Clinicians should be wary of unspecific symptoms, such as pain or elevated levels of ALP and/or LDH, as they may suggest bone and bone marrow involvement. However, these markers are not always abnormally elevated and further tests are generally required [5,8,20]. The diagnosis may be established by imaging methods, such as CT, magnetic resonance, bone scintigraphy, and positron emission tomography, and by biopsy [8,15,16,18]. Often, the pain has a marked impact on a patient's quality of life and is usually managed with opioids [11], surgery, or radiotherapy [8,20]. Chemotherapy may be offered for GC patients with bone or bone marrow metastases, however, due to the possible co-existence of cytopenia and deterioration of the patient's performance status, it may be challenging and a standard regimen has not been established [5,8,11,16]. Bisphosphonates or denosumab may decrease solid tumor skeletal-related events, however, prospective trials are still needed in GC, as data on this neoplasia is scarce [8,11,18].

## Conclusions

Although infrequent, GC should be considered in the differential diagnosis of a patient with secondary bone lesions, as it generally may not be the first hypothesis. The clinical features of GC with bone metastases are still not completely understood, and its diagnosis and treatment are still suboptimal. Therefore, these topics should be the target of further prospective studies and trials in order to anticipate early diagnosis and improve therapeutics, pain control, and quality of life.
